# Photoemission Study of GaN Passivation Layers and Band Alignment at GaInP(100) Heterointerfaces

**DOI:** 10.1021/acsami.4c17453

**Published:** 2025-01-14

**Authors:** Sahar Shekarabi, Mohammad Amin Zare Pour, Haoqing Su, Wentao Zhang, Chengxing He, Kai Daniel Hanke, Oleksandr Romanyuk, Agnieszka Paszuk, Wolfram Jaegermann, Shu Hu, Thomas Hannappel

**Affiliations:** † Grundlagen von Energiematerialien, Institut für Physik, Technische Universität Ilmenau, 98693 Ilmenau, Germany; ‡ Department of Chemical and Environmental Engineering, Yale University, New Haven, Connecticut 06520, United States; § FZU - Institute of Physics of the Czech Academy of Sciences, Prague 16200, Czech Republic; ∥ Surface Science Laboratory, Department of Materials and Earth Sciences, Technische Universität Darmstadt, 64287 Darmstadt, Germany

**Keywords:** solar energy conversion, photoelectrochemical cell, MOVPE, band alignment, III−V semiconductor, GaN, passivation layer

## Abstract

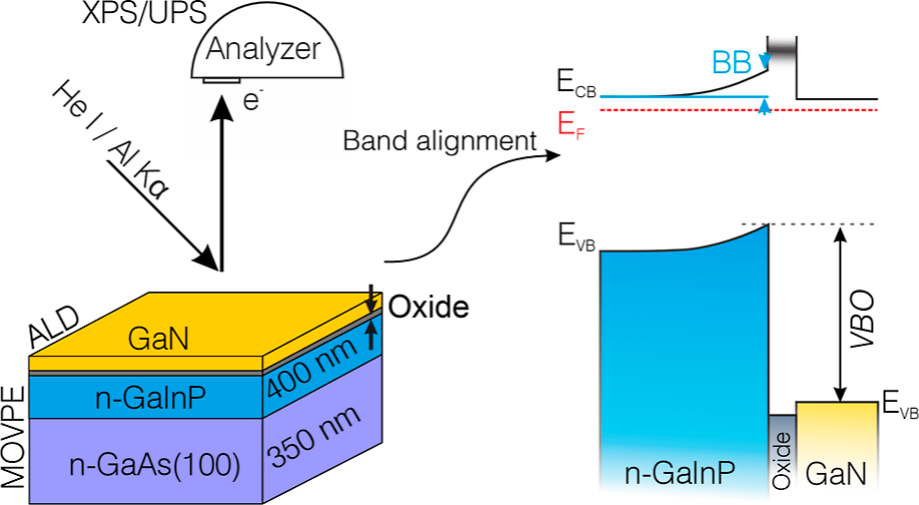

To date, III–V semiconductor-based tandem devices with GaInP top photoabsorbers show the highest solar-to-electricity or solar-to-fuel conversion efficiencies. In photoelectrochemical (PEC) cells, however, III–V semiconductors are sensitive, in terms of photochemical stability and, therefore, require suitable functional layers for electronic and chemical passivation. GaN films are discussed as promising options for this purpose. The band alignment between such a protection layer and the III–V semiconductor should be aligned to minimize corrosion and nonradiative interfacial recombination and to promote selective charge carrier transport. Here, we investigate the band alignment between GaN passivation layers and n-type doped GaInP(100) photoabsorbers and grew n-type GaInP(100) epitaxially by metalorganic vapor phase epitaxy on oxidized GaAs(100) substrates to mimic a realistic preparation sequence. We prepared 1–20 nm GaN films on top employing atomic layer deposition and studied the band alignment at the GaN/GaInP(100) heterointerface by X-ray and ultraviolet photoelectron spectroscopy. Due to the limited emission depth of photoelectrons, we determined the band alignment by a series of measurements, in which we increased the thickness of the GaN films successively. The n-GaInP(100) surfaces, prepared with a well-known phosphorus-terminated p(2 × 2)/c(4 × 2) reconstruction, show an upward surface band bending (BB) of 0.38 eV and a Fermi level pinning due to the present surface states. Upon oxidation, the surface states are partially passivated, resulting in a reduction of the BB to 0.16 eV and a valence band offset (VBO) between the GaInP(100) and the thin oxide layer of 2.01 eV. Applying Kraut’s approach, we identified a VBO of 1.90 eV and a conduction band offset of 0.44 eV between GaInP(100) with a thin oxide layer and the GaN passivation layer. We conclude that the GaN is a well-suited passivation layer for PEC cells and facilitates selective transport of photogenerated electrons.

## Introduction

The quest for sustainable energy sources has driven extensive research into semiconductor materials as photoelectrochemical (PEC) cells for direct unassisted solar-driven water splitting, CO_2_ reduction, and higher-value fuel generation.^1−4^ Among various semiconductors, III–V compounds play a crucial role in a wide range of electronic and optoelectronic devices, including diode lasers, light-emitting diodes, photodiodes, optical modulators, and multijunction photovoltaic or PEC devices.^1,5,6^ Ternary compounds, in particular, offer the ability to precisely adjust their band gap and their lattice constant, which allows a flexibility of forming and fine-tuning well-aligned III–V heterojunctions and to precisely control their electronic properties.^7^ Therefore, the harvest of solar radiation can be boosted by an application of several III–V photoabsorbers with different band gaps (multijunction devices), which results in higher solar-to-electricity or -hydrogen conversion efficiencies, compared to traditional silicon-based systems.^8,9^ Such III–V multijunction devices recently achieved remarkable solar-to-electricity and solar-to-hydrogen conversion efficiencies above 47%^5,10^ and above 19%,^6^ respectively.

Ga_0.51_In_0,49_P with a band gap of around 1.84 eV (lattice matched to GaAs) is an excellent choice for use as a top photoabsorber in highly efficient tandem structures^6,11,12^ with the theoretical solar-to-hydrogen conversion efficiency surpassing 20%.^13−15^ However, Ga_*x*_In_1–*x*_P faces significant challenges related to corrosion and its durability when exposed to aqueous solutions and light.^15,16^ Surface corrosion can lead to increased recombination losses or even decomposition of the material, which significantly affects the device performance. Thus, its suitability for immersed operation in a PEC setup is critical.^16,17^ Another factor detrimental to the performance of the PEC cells is the native oxides formed on the III–V materials. The interfaces between III–V semiconductor compounds and their native oxides are often compromised by defects.^18,19^ So far, for III–P semiconductors, various surface cleaning techniques and their effect on the band alignment toward the native oxide layer have been studied.^20−22^ To address this challenge, various strategies have been applied to improve the performance, stability, and viability of Ga_*x*_In_1–*x*_P for its integration into PEC devices, such as utilization of appropriate protection layers as well as chemical and electronic surface passivation.^6,15,23^ For example, Gu et al. achieved stable operation of p-type GaInP/TiO_2_/cobaloxime for 20 min,^15^ while Lim et al. demonstrated stability for up to 124 h by depositing MoS_2_ on GaInP.^24^ Passivation layers must have larger band gaps than the top photoabsorber to prevent undesirable absorption of solar radiation. AlInP is successfully utilized as a passivation layer with the highly effective GaInP top absorber, which is used in various record device structures for both photovoltaic and PEC applications.^1,6,25,26^ Consequently, the band alignment at the GaInP/AlInP heterointerface has been studied thoroughly.^6,27^ However, due to the stability issues that III–V materials, including AlInP, face in corrosive environments, GaN is considered as an alternative charge selective contact layer and protective layer in one. GaN due to its wide band gap (approximately 3.4 eV) and thus its negligible sunlight absorption can serve as an excellent passivation layer for the top III–V photoabsorber used in PEC devices.^28−33^ Compared to III–As and III–P compounds, III-nitrides, like GaN, have stronger ionic chemical bonds, making them more stable over a wider pH range.^30,34,35^ Moreover, compared to the TiO_2_ protection layer, GaN has higher stability under a wider range of applied bias.^36−40^

The electronic structure at the heterointerface between the n-GaInP absorber and the GaN passivation layer (here, also acting as the protection layer) is of crucial importance as it determines the complex charge carrier dynamics at the interface, i.e., the selective transport of charge carriers as well as the interfacial recombination of photogenerated electron–hole pairs.^35,41,42^ Only a complete band energy diagram, which is the focus of the work presented here, can give an understanding of the properties of the entire device structure.

A large conduction band offset (CBO) leads to low photocurrent, and the reduced conductivity for electrons hinders their selective transport, which is essential for an optimized device performance.^41−44^

The determination of valence band offsets (VBOs) through Kraut’s method using X-ray photoelectron spectroscopy (XPS) has emerged as a prominent approach in characterizing semiconductor materials.^45^ Through analysis of core level (CL) and valence-band spectra, the energy alignment and band offset between different materials can be accurately assessed. Kraut’s method considers the effect of space charge layers due to charge carrier displacement across the interface of two materials. First, the binding energy of the high-intensity CL and the valence band maximum (VBM) are considered for both materials. Subsequently, data are obtained from the samples on which the first material is coated with a thin film of the second material. It is crucial that the thickness of the thin film is sufficiently small to allow the photoelectrons from the underlying layer to escape and be detected during the XPS measurement. This allows precise interface-sensitive measurements in which photoemission (PE) peaks from both materials are resolved.^45,46^

The band diagram will strongly differ depending on the type of the semiconductor (here, the n-type GaInP photoelectrode) and its surface preparation prior to atomic layer deposition (ALD) deposition. It should be noted that determination of the VBO only would not be sufficient to understand the properties of the final device structure. Still, a complete band energy diagram is needed, which is the main aim of the given work.

Here, the band alignment between the oxidized n-type GaInP(100) photoabsorber and the GaN passivation layer was studied using photoelectron spectroscopy, and Kraut’s method was applied to multiple successive heterojunctions to draw the whole picture of the GaInP/oxide/GaN band alignment.

## Experimental Section

The n-type Ga_0.51_In_0.49_P(100) (hereinafter referred to as n-GaInP) epitaxial layers were grown on the n-type GaAs(100) substrates, misoriented by a 0.1° offcut in the [111] direction, in a H_2_-based horizontal-flow metalorganic vapor phase epitaxy (MOVPE) reactor (Aixtron, AIX-200). The entire process was monitored in situ by surface sensitive reflection anisotropy spectroscopy (RAS, LayTec).^47,48^ The GaAs(100) substrates were deoxidized at 620 °C under tertiary-butylarsine (TBAs) precursor flow for 10 min^49^ Subsequently, at the same temperature, a 100 nm thick GaAs(100) buffer layer was grown with a molar V/III ratio of 21.2 using TBAs and triethylgallium (TEGa). The process was followed by an epitaxy of a 400 nm thick n-GaInP layer with a molar V/III ratio of 32.6, at 600 °C and a pressure of 100 mbar, utilizing TEGa, tertiary-butylphosphine (TBP), and trimethylindium. Di-tertiary-butyl silane (DTBSi) was used as the n-type dopant source, with the molar flow adjusted to achieve carrier concentrations of approximately 4.0 × 10^18^ and 2.0 × 10^16^ cm^–3^ in the GaAs and the GaInP layers, respectively.

The GaInP(100) growth rate of 0.24 nm/s was determined on a reference sample by in situ RAS measurements of the periodic Fabry–Pérot oscillations.^50^ After the growth of the GaInP(100) layer, the sample was cooled to 300 °C under the flow of the TBP precursor. Subsequently, a phosphorus-terminated (P-rich) p(2 × 2)/c(4 × 2) surface reconstruction was prepared at 310 °C without supply of the TBP precursor.^51,52^ During the surface preparation, RA spectra were measured continuously until the RAS signal reached a characteristic RAS line-shape with a stable and maximized amplitude.^53^ Electrochemical capacitance–voltage profiling (ECVP, WEP-CVP 21) was used to determine the carrier concentration depth profile in the III–V epilayers (*ex situ* with 0.1 M HCl solution, under visible light illumination at room temperature).^54^ Results of the carrier concentration are shown in the Supporting Information in Figure S1.

Ex situ high-resolution X-ray diffractometry (HR-XRD) scans (Bruker AXS D8 Discover with Ge(022)x4 asymmetric monochromator and Goebel mirror, copper alpha line = 1.5405 Å) were performed to confirm that the lattice constant of the GaInP(100) epilayer is identical with the lattice constant of the GaAs(100) substrate (*a* = 5.654 Å). Based on the XRD results, the atomic stoichiometry of the Ga_*x*_In_1–*x*_P bulk was determined to be *x* = 0.51, which corresponds to the lattice constant of *a* = 5.654 Å.^55^ The HR-XRD data are included in the Supporting Information, Figure S2. To further characterize the crystallinity of the ALD-grown GaN layers, additional XRD measurements were conducted on a sample with 30 nm of ALD-grown GaN on a GaInP/GaAs substrate with its (100) edge parallel to the X-ray beam plane. In Figure S3a, the θ – 2θ scan result shows peaks from the diffraction of the GaAs and GaInP. To minimize the signal from the substrate, grazing incidence X-ray diffraction (GIXRD) was performed at an incident angle of ω = 0.313, yielding a diffraction pattern specific to the GaN coating. The GIXRD (Figure S3b) indicates the presence of diffraction patterns from different GaN planes, demonstrating that the ALD-grown GaN coating exhibits a well-defined polycrystalline structure.

Selected samples were transferred from the MOVPE reactor to the ultrahigh-vacuum (UHV) analytical chamber by a UHV transfer system with a pressure below 5 × 10^–10^ mbar.^56^ The analytical chamber is equipped with XPS (monochromated Al Kα line, Specs Focus 500/Phoibos 150/1D-DLD-43-100), ultraviolet photoelectron spectroscopy (UPS, HeI, Specs Focus 500/Phoibos 150/1D-DLD-43-100), and low-energy electron diffraction (LEED, Specs ErLEED 100-A),^56^ where the LEED pattern was recorded using a primary electron beam energy of 63 eV.

Prior to the deposition of GaN films, the air-exposed surface of the n-GaInP was etched using a commercial 1.25 M solution of HCl in 2-propanol (Aldrich) for 1 min to eliminate weakly bounded surface contaminant species.^57^ Etching the n-GaInP surfaces with only a 1 M aqueous HCl solution was also studied but not applied here as it was less effective in removing the oxide and led to a significant quantity of phosphates. Ar-ion sputtering was not applied here since it can change the chemical composition of the surface.^21^

GaN layer deposition was performed by using a commercial ALD system (Ultratech Fiji G2). Following the etching of oxidized n-GaInP, the samples were loaded into the ALD system. Trimethylgallium (TMGa, Strem Chemicals, 99.9999%-Ga) was used as the gallium precursor, and N_2_ plasma served as the nitrogen precursor. Ultrahigh purity grade argon and nitrogen gas tanks (Airgas, Inc.) were used as the gas supply for the ALD system. The temperatures of the TMGa precursor cylinder, the delivery line, and the susceptor were maintained at 25, 150, and 350 °C respectively. As shown in Figure S4, each ALD cycle consisted of a Ga dose and a N dose with 30 sccm Ar flow as the carrier gas. In the Ga dose, a 0.015 s pulse of TMGa with 80 sccm Ar and 5 sccm N_2_. During this step, the plasma generator remained deactivated and the turbo pump was disabled. Subsequently, in the 60 s N dose, the turbo pump starts to connect to the reaction chamber with 200 sccm Ar and 40 sccm N_2_ through the plasma generator, and the plasma generator is activated three times at 300 W for 20 s each time. During the Ga dose and N dose, the process pressures are 0.16 mbar and 0.04 mbar, respectively, due to the status change of the plasma flow rate and turbo pump. According to the atomic percentage calculated based on XPS measurements (shown in Figures 4a and S6), the Ga to N ratio is approximately 1.03, suggesting an almost 1:1 ratio between Ga and N. The thickness of the GaN layers was determined using both the cumulative number of performed ALD cycles as well as ellipsometry: 1, 3, 5, 10, and 20 nm were deposited by 20, 60, 100, 200, and 400 ALD cycles, respectively.

XP spectra were measured with respect to the calibrated Fermi level by using sputtered Au, Ag, and Cu reference samples. Spectra were acquired using two photoelectron emission angles: (i) the initial measurements were conducted at a standard emission angle of 90° between the detector and the sample and (ii) at 30° between the detector and the sample by tilting the sample to enable a more surface-sensitive analysis. The survey scan and high-resolution CL spectra were measured with a pass energy of 30 and 10 eV, with an energy step of 0.5 and 0.05 eV, respectively. UPS He I spectra were measured with an energy step of 0.05 eV. We used SpecsLab2 and CasaXPS software to analyze and fit each XP spectrum and a Lorentzian function to fit the high-resolution CL spectra. For the CL fits, Lorentzian asymmetric line shapes with varying Lorentzian-to-Gaussian ratios were used: In 3d_5/2_ and N 1s peaks were fitted with an LA(75) line shape, while P 2p, Ga 2p_3/2_, and O 1s peaks were fitted using an LA(50) line shape. Using the corresponding fitted PE areas and the relative sensitivity factor, the chemical composition of the film was quantified.^58,59^

## Results and Discussion

### Analysis of the Band Alignment near the P-Rich GaInP(100) Surface

**Figure 1:**
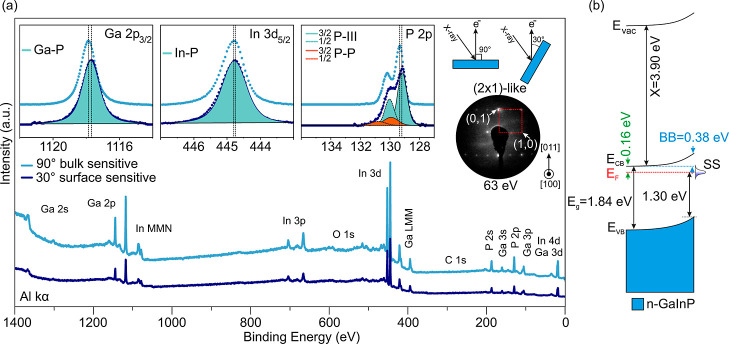
(a) Survey and high-resolution XP spectra of the P-rich n-GaInP(100) surface. The inset shows the corresponding LEED pattern, which exhibits a (2 × 1)-like surface reconstruction. Above the LEED pattern, a schematic picture of the experimental setup is shown. The top row shows high-resolution spectra of Ga 2p_3/2_, In 3d_5/2_, and P 2p (from left to right) with corresponding fitted data of 30° measurement. The light blue spectra represent measurements at 90°, while dark blue represents measurements at 30°. All spectra are shown after the subtraction of the background. (b) Sketch of the band diagram near the P-rich n-GaInP(100) p(2 × 2)/c(4 × 2) surface.

Figure 1a shows XP spectra and a LEED pattern (inset) measured on the as-prepared, P-rich n-GaInP surface. The LEED pattern, taken at primary electron beam energies of 63 eV, shows half-order spots and diffuse half-order streaks along the [011] direction, characteristic for a P-rich (2 × 1)-like pattern, which is a superposition of p(2 × 2) and c(4 × 2), surface reconstruction.^27^ Analogous LEED patterns were formerly observed on P-rich GaP(100) and InP(100) surfaces,^60^ as well as on other ternary compound surfaces such as P-rich AlInP(100).^27^ These surfaces consist of arrays of buckled P–P dimers, each passivated with one hydrogen atom. The saturation of P–P dangling bonds by one H atom induces arrangements of p(2 × 2) and c(4 × 2) surface unit cells—the origin of diffuse streaks in the LEED pattern.^60^ On an ideal surface, all P–P dimers are passivated by one hydrogen atom. However, it was observed that hydrogen might be missing at P–P dimers, which leads to surface states in the midgap and causes pinning of the Fermi level.^61^ Moritz et al. have extensively discussed such defects on the P-rich InP(100) surface, taking into account variations in temperature and pressure during preparation in MOVPE.

The XPS survey spectra of both 90 and 30° measurements provide clear evidence that there is no contamination present on the surface (see Figure 1a). Furthermore, high-resolution XP spectra of O 1s and C 1s (not shown here) confirm that the sample was not contaminated during the UHV transfer from the MOVPE reactor to the XPS chamber. The P 2p CL is fitted with two spin–orbit pairs, as shown in Figure 1 (top row, right): The dominant pair corresponds to the contribution from the bulk (light blue), and the smaller component, shifted to higher binding energies by 0.79 eV (orange), is related to the P–P dimers on the surface.^62^ The surface component becomes larger with the 30° emission angle. In contrast to the P 2p, both Ga 2p_3/2_ and In 3d_5/2_ CL peaks were fitted with only one component related to the bulk, Ga–P and In–P bonds, respectively.

**Figure 2:**
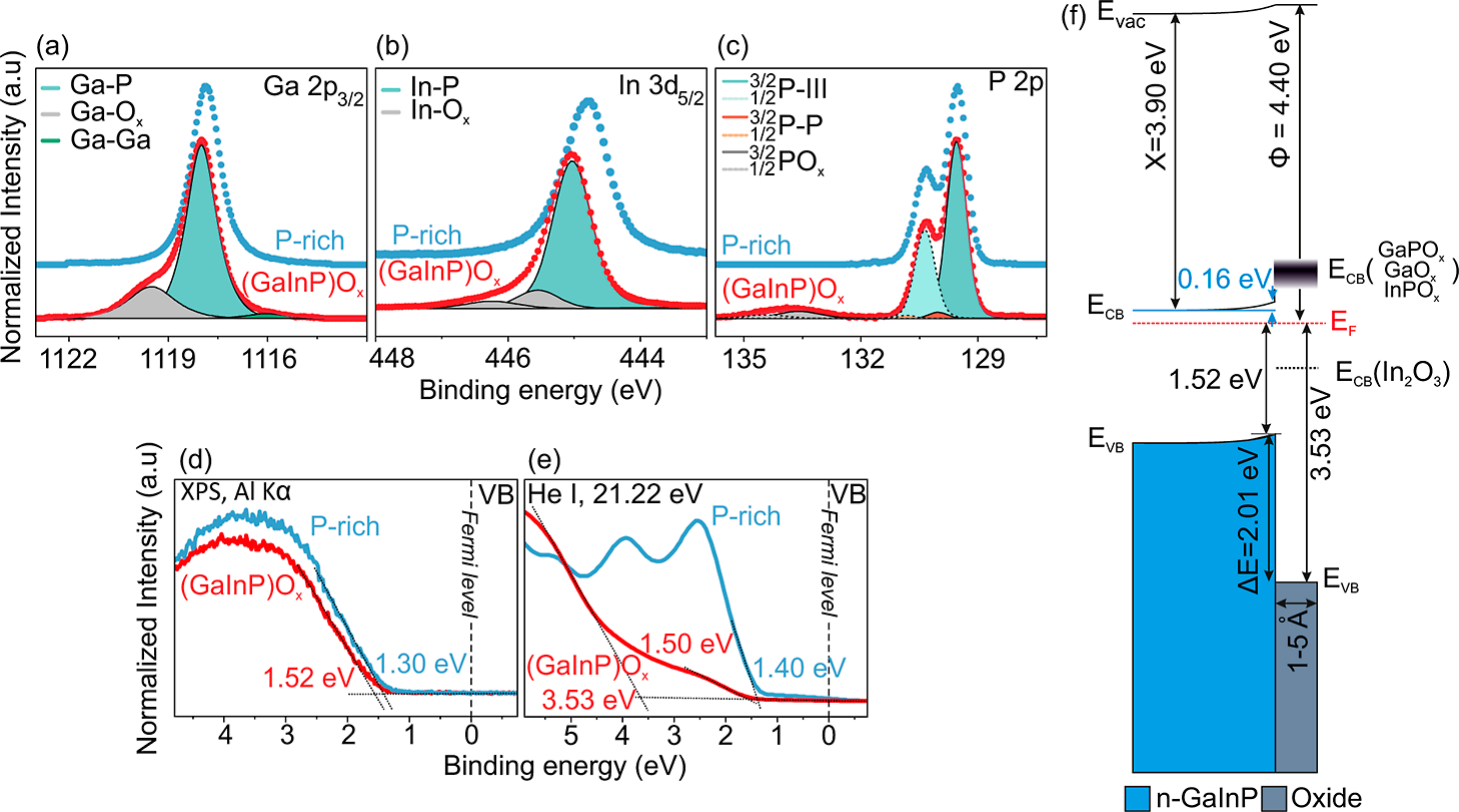
Measured CL spectra of (a) Ga 2p_3/2_, (b) In 3d_5/2_, and (c) P 2p after background subtraction on P-rich n-GaInP (blue) and etched (GaInP)O_*x*_ (red) samples. (d) XPS and (e) UPS (He I) of the valence band. (f) Band diagram of n-GaInP after etching ((GaInP)O_*x*_).

Based on the XPS results of the P-rich n-GaInP sample, the VBM is found to be at 1.30 eV below the Fermi level position; see Figure 2d. With the GaInP band gap determined by spectral ellipsometry to be 1.84 eV,^63−65^ the Fermi level position at the surface is located slightly above the midgap. The difference between the conduction band minimum (CBM) and the Fermi level, *E*_F_–*E*_CB_, is , where the effective density of states and density of electrons in the conduction band are denoted by *N*_C_ and *n*_e_, respectively.^66^ (see eq 1)
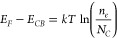
1Here, *N*_C_ is estimated to be 9.8 × 10^18^ cm^–3^, taking the average of the values for GaP and InP.^67^ The *n*_e_ is estimated to be the dopant concentration, which is approximately 2.0 × 10^16^ cm^–3^ based on ECVP measurements (see Supporting Information, Figure S1). Therefore, the Fermi level in the bulk is expected to be 0.16 eV below the CBM. The difference between the measured and calculated Fermi level positions relative to the valence band indicates a strong surface band bending (BB) of 0.38 eV due to the presence of surface states, as shown in Figure 1b. Changing the photoelectron emission angle from 90 to 30° induced a shift of all CL peaks toward lower binding energies, which confirms upward BB toward the surface. Figure 1b shows the band diagram near the surface of P-rich n-GaInP.

### Analysis of the Oxidized n-GaInP(100) Surface

The air-exposed P-rich n-GaInP(100) surface was wet chemically etched as described in the Experimental Section to reduce surface contaminant species and the oxide layer thickness (see Supporting Information, Figure S5).^57^Figure 2 compares the (a) Ga 2p_3/2_, (b) In 3d_5/2_, and (c) P 2p CL spectra of the as-prepared P-rich n-GaInP surface (light blue spectra, the same as in Figure 1) with the etched ((GaInP)O_*x*_) surface (red spectra), measured at 90° angle. Upon comparison of the corresponding CL spectra of those two samples, changes in the line-shape, as well as a CL shift to a higher binding energy, were observed: The spectra of the oxidized samples include contributions from the oxide components (gray). Although the specific types of oxides are not specified, a general peak represents the combined contributions of all of the oxides in the fittings. One of the crucial oxide species in PEC application is In_2_O_3_ with a band gap of 2.90 eV.^68^ This oxide species serves as a trap for charge carriers. The presence of this species is typically observed in the O 1s CL at an energy of 530 eV. We resolved the presence of In_2_O_3_ on the oxidized GaInP sample. However, after growing GaN, the presence of In_2_O_3_ was no longer detectable (see Supporting Information, Figure S7k).The shifts correspond to a reduction in the upward surface BB from 0.38 to 0.16 eV. Thus, the change in the BB indicates a change in charged states on the surface, implying a partial passivation of the surface states.

In addition to the oxide components, on the oxidized sample, the P 2p spectrum of the oxidized sample shows a small surface component (orange line) at around 129.8 eV can be fitted (Figure 2c). This component could potentially be attributed to the presence of residuals of P–Cl bonds (around 3% Cl) caused by etching in HCl acid or could also be attributed to residuals of the P–P bonds on the surface.^20^

Figure 2d,e shows the VB spectra measured by XPS and UPS, respectively, of both the etched (red spectrum) and as-prepared P-rich n-GaInP (blue spectrum) surfaces. The information depth of the VBM measured by UPS (VB-UPS) (around 1–2 nm) is smaller than the information depth of the VBM measured by XPS (VB-XPS) (5–10 nm). Thus, the utilization of UPS can be employed to investigate the electron emission originating from the native oxide layer, while in the case of VB-XPS, the emission from the GaInP layers superimposes with the emission from the oxide layer.^20^ The VBM of the native oxide on the n-GaInP layer is approximately 3.53 eV (Figure 2e, red curve), while the VBM of n-GaInP based on both XPS and UPS measurements is 1.52 eV.

Based on the measurements of the VBM, the band diagram of the oxide/n-GaInP was constructed as shown in Figure 2f. The band gap of the oxide layer was assumed to be between 4.50 and 4.80 eV, based on the values from the literature, including GaPO_4_, InPO_4_, and PO_*x*_.^67^ Consequently, the lower and upper limits of the conduction band minima are illustrated with the band gaps of the different species present in the oxide layers (gradient gray square, *E*_CB_). The thickness of the native oxide layer was determined to be ca. 1–5 Å^6,69^ (see Supporting Information, Table S1). Considering the *E*_F_–*E*_CB_ of −0.16 eV and VBM of 1.52 eV, the BB at the n-GaInP surface decreased after oxidation from 0.38 to 0.16 eV. Note that the absence of BB within the oxide layer is regarded as a reasonable assumption due to its small thickness and probably the limited presence of mobile charge carriers.^20^ We derived a VBO of 2.01 eV between the oxide and the n-GaInP layer. In the following, the oxidized n-GaInP samples were used as substrates for the growth of the GaN layers by ALD.

### Analysis of the GaN/Oxide/GaInP(100) Heterointerface

**Figure 3:**
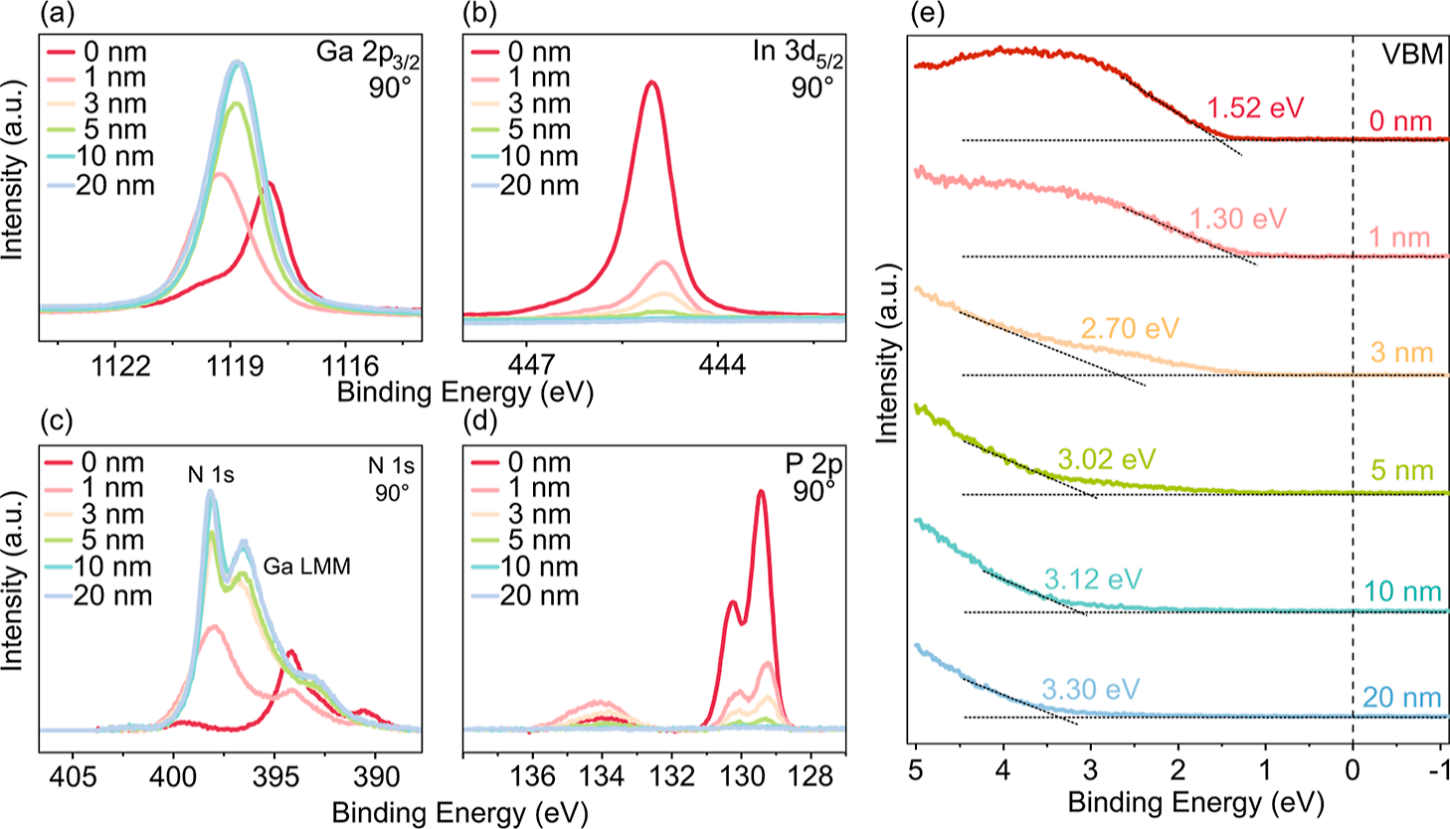
CL spectra of (a) Ga 2p_3/2_, (b) In 3d_5/2_, (c) N 1s, (d) P 2p, and (e) VBM with respect to the thickness of the GaN overlayer.

A series of samples with different thicknesses of GaN layers ranging from 1 to 20 nm were prepared by ALD on the n-GaInP/n-GaAs(100) samples and analyzed by XPS and UPS. The XP spectra of every main CL across all GaN samples are displayed in Figure 3a–d. As expected, upon increasing the thickness of the GaN layer, the intensities of the In 3d_5/2_ and P 2p CL peaks decreased, while the N 1s intensity increased (note that the peak at 394.46 eV in Figure 3c for the 0 nm sample is the Ga LMM Auger peak^70^). These changes in intensity are due to an increase in the amount of GaN content on the surface, accompanied by a decrease in the photoelectron emission from the n-GaInP epilayer.

**Figure 4:**
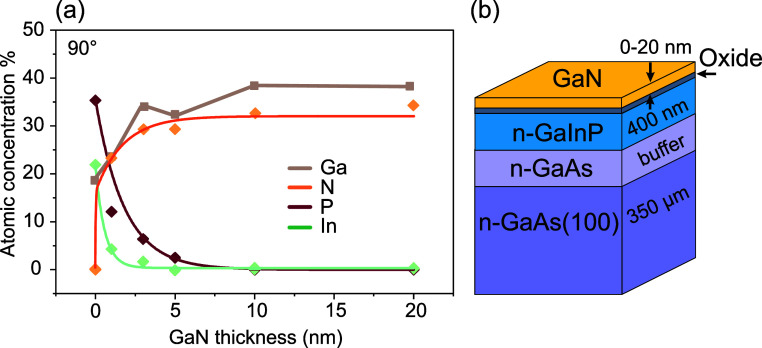
(a) Atomic concentration of N, Ga, In, and P in the bulk of GaN/n-GaInP samples for 0–20 nm thick GaN overlayers. (b) Schematic view of the GaN/oxide/n-GaInP sample.

Quantification of the samples’ atomic composition is shown in Figure 4a, which was determined by utilizing the fitted PE areas and the respective relative sensitivity factor.^59^ The concentrations of Ga and N increased almost exponentially with the increase of the thickness of the GaN layer. Fluctuations in the Ga concentration and GaN stoichiometry are visible, in particular, at the beginning of interface formation, which can be attributed to the presence of the thin oxide layer on the n-GaInP surface. It should also be noted that oxygen and carbon are not shown in this plot but are included in the Supporting Information, Figure S5. As expected, the concentrations of In and P decreased in parallel to the increase of the GaN thickness. The In concentration dropped more rapidly than the P atomic concentration, which could be related to the higher thickness of the P-based oxides than that of the In oxides. This indicates the formation of a P-containing intermediate layer at the interface rather than an intermediate layer with In.

The Ga 2p_3/2_ peak in Figure 3a shows a strong shift of the maximum as we start depositing the GaN. This shift is attributed to a chemical shift related to the contribution of the oxide/phosphate intermediate layer (GaO_*x*_/GaPO_4_), which is particularly evident at lower GaN deposited layer thicknesses. It is important to note that the very first layers of GaN may differ from the thicker layers due to different initial nucleation and growth conditions during the early stages of deposition. These differences may be associated with defects formed during interface formation, which can lead to slight modifications of the energy band alignment. These interface modifications can lead to chemical shifts in each element of the CLs in XPS data (see Figure 3).^71−73^ However, these variations do not significantly impact the overall band alignment, which is defined across all of the entire heterostructures. For thicker layers of stoichiometric GaN, the alignment of the Fermi levels of GaN and n-GaInP dominates, shifting the Ga 2p_3/2_ emission again to lower binding energies due to the VBO and a back shift of BB on the n-GaInP substrate induced by the different doping level between the two semiconductors with different band gaps. On the samples with a low GaN thickness (1–3 nm), in the Ga 2p_3/2_ peak, we expect contributions from both the overlayer (GaN) and the substrate (GaInP). Consequently, the PE peak position may change due to varying contributions of the overlayer (GaN) and the substrate (GaInP) at the initial stages during the formation of the heterointerface. However, the Ga 2p_3/2_ and N 1s peak positions remained almost constant on the samples with GaN thicknesses above 3 nm. A slight binding energy shift of 0.20 eV to lower binding energies was observed in the In 3d_5/2_ and the P 2p CLs on the samples covered with GaN layer thickness between 0 nm and >1 nm. This shift suggests a possible charge carrier transfer between the GaN overlayer and the n-GaInP substrate, which could affect the initial oxide/n-GaInP surface BB. A similar effect was observed during the oxidation of the P-rich n-GaInP surface, as discussed earlier.

**Figure 5:**
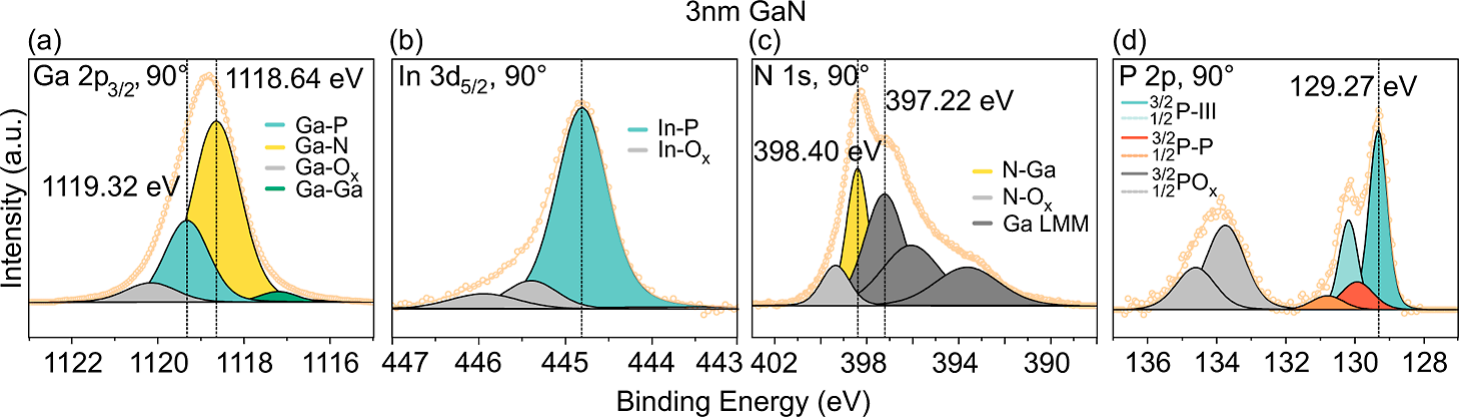
XP spectra of (a) Ga 2p_3/2_, (b) In 3d_5/2_, (c) N 1s, and (d) P 2p of the 3 nm GaN/n-GaInP sample.

**Figure 6:**
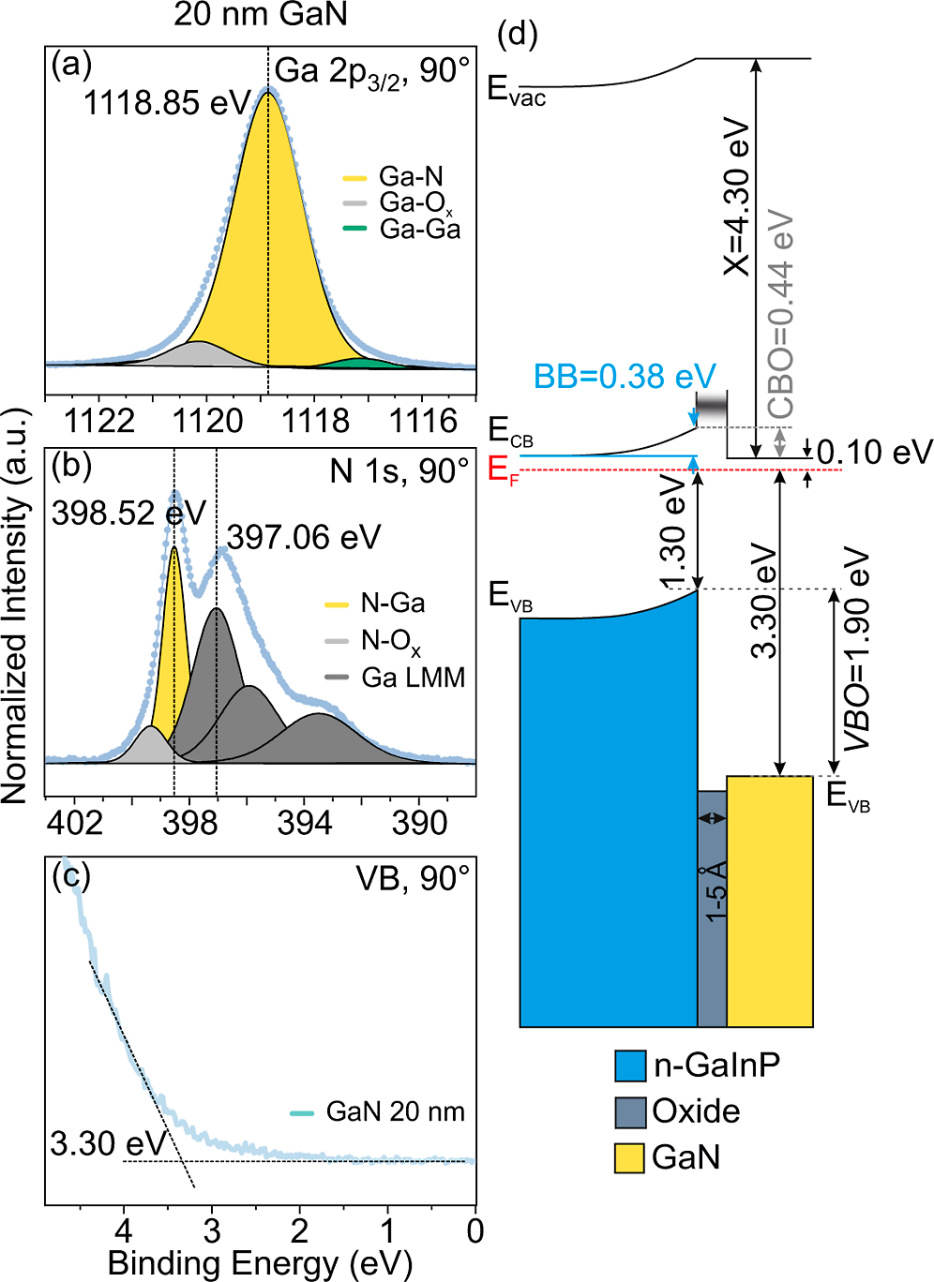
XPS of (a) Ga 2p_3/2_ and (b) N 1s CL spectra and (c) VBM of 20 nm GaN/n-GaInP. (d) Band diagram of n-GaInP/oxide/GaN.

Figures 5 and 6 show peak fitting of the CL spectra that are measured on a thin (3 nm) and a thick (20 nm) GaN/oxide/n-GaInP heterostructure, respectively. We can consider the sample with a 20 nm GaN film as only bulklike since no signal from the n-GaInP substrate is expected according to the mean inelastic free path of electrons in GaN. In Figure 5a, the Ga 2p_3/2_ peak of the 3 nm sample contains various contributions to the PE intensity: a Ga–N component at 1118.64 eV, the n-GaInP substrate (Ga–P component) at 1119.32 eV, and contributions from the oxide and Ga–Ga bonds. The chemical shift of the oxide component is 1.50 eV toward higher binding energies, and for Ga–Ga, it is 1.40 eV toward lower binding energies.

The N 1s spectra of both samples with 3 and 20 nm GaN contain contributions from the N–Ga bonds, Ga LMM Auger peaks,^74^ and N–O_*x*_ bonds. It is noteworthy that the binding energy difference between the Ga–N-related components reflected in the Ga 2p_3/2_ and N 1s peaks is almost the same for both 3 and 20 nm: 720.24 eV (1118.64–398.40 eV) for the 3 nm sample and 720.33 eV (1118.85–398.52 eV) for the 20 nm sample. Therefore, the Ga–N component of GaN can be unambiguously identified in the Ga 2p_3/2_ peak in Figure 5a.

The In 3d_5/2_ peak of the 3 nm sample consists of a component related to In–P and In–O_*x*_ bonds, as shown in Figure 5b. The P 2p peak includes contributions from the P–Ga(In) bonds of the substrate (light blue), P–P or P–Cl surface components (orange),^20^ and a pronounced oxide component P–O_*x*_ (gray).

Therefore, by observing these oxide contributions in each CL for the GaN samples with a thickness of 1–5 nm (shown in Figures 5 and S7a,j), we confirmed the presence of the intermediate oxide layer at the GaN/n-GaInP interface. Based on XP spectra of the 10 and 20 nm GaN samples, we conclude that there are no contributions from In or P atoms for the thicker GaN samples in the XP spectra. The PE peak positions of all CLs are summarized in Table 1. Comprehensive data fitting for all CLs of the samples with 1–10 nm GaN/n-GaInP can be found in the Supporting Information, Figure S7.

**Table 1:** Measured Binding Energies and VBOs on *n*-GaInP/GaN by XPS

thicknesses (nm)	peak position (eV)						VBM (eV)	VBO (by P 2p_3/2_)	VBO (by In 3d_5/2_)
	P 2p_3/2_	In 3d_5/2_	Ga 2p_3/2_		N 1s	Ga LMM			
	(P–III)	(In–P)	(Ga–P)	(Ga–N)	(N–Ga)				
0	129.44	445.03	1118			394.46			
1	129.28	444.87	1119.43	1118.85	398.35	397.12	1.30	1.82	1.82
3	129.27	444.86	1119.32	1118.64	398.40	397.22	2.70	1.88	1.88
5	129.31	444.88	1119.52	1118.79	398.49	397.07	3.02	1.93	1.95
10				1118.77	398.38	397.04	3.12		
20				1118.85	398.52	397.06	3.35		

The VBO, denoted as Δ*E*_VB_, was calculated using Kraut’s equation,^45,75^ incorporating the CL peak positions as follows (Kraut’s equation)
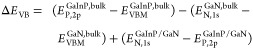
2

In eq 2, three terms were considered. The first term (*E*_GaInP, bulk P, 2p_ – *E*_GaInP, bulk, VBM_) represents the energy difference between the PE CL (P 2p) and the VBM of the n-GaInP sample (without GaN). The second term (*E*_GaN, bulk, N 1s_ – *E*_GaN, bulk, VBM_) represents the energy difference between the PE CL (N 1s) and the VBM for the 20 nm GaN sample. The third term (*E*_GaInP/GaN, N 1s_ – *E*_GaInP/GaN, P 2p_) includes the CL energy difference measured in 1–5 nm GaN/oxide/n-GaInP heterostructures. We used two sets of CL pairs for VBO calculations: the P 2p CL of the GaInP layer (also calculated with In 3d_5/2_, see Table 1) and N 1s CL of the GaN overlayer. The results of these calculations are included in titer 1, and the determined VBO values between the oxidized n-GaInP substrate and the GaN overlayer are 1.90 ± 0.10 eV.

The resulting band diagram of the n-GaInP/oxide/GaN heterostructure is illustrated in Figure 6d. Based on the measured VBO value of 1.90 eV, the VBM at 3.30 eV (see Figure 3e), and the known band gap of the GaN (3.40 eV),^28^ the CBO was determined to be 0.44 eV, indicating that the CBM of the GaN is below the CBM of the n-GaInP layer. This suggests a nearly barrier-free pathway for electron transport across the interface, which is ideal for selective transport of electrons. In addition, there is a significant VBO, forming a barrier, which substantially reduces the conductivity for holes.^41^

The presence of an oxide layer in the heterostructure is confirmed by the observed oxide contribution at the PE CLs, as depicted in Figure 5. According to the calculations, the oxide layer at the heterointerface has a thickness between 1 and 5 nm. This is thin enough to enable electron tunneling, similar to in the tunnel oxide passivated contact solar cell,^76,77^ thereby facilitating the movement of the electrons across the interface. The observed reduction in the VBM of n-GaInP to 1.30 eV after the growth of GaN by ALD indicates an increase in the degree of BB. Identifying the precise ingredient responsible for this increase is complicated due to the presence of overlapping constituents at the CLs, including Ga–N and Ga–O. This implies that ongoing chemical interactions occur with the oxide layer throughout the ALD process, leading to alterations in its chemical composition. The comprehensive band energy diagram reveals that the electronic structure of the GaN/oxide/n-GaInP heterointerface could be well-suited as a photocathode for efficient hydrogen evolution reactions.^78^ For that, it is crucial to have electron selective transport through contact with a favorable alignment of the electronic band structure to the adjacent semiconductor. This alignment ensures that the energy levels of the contact in the conduction band closely match those in the adjacent semiconductor. With such an alignment, electron currents can flow selectively between the contact and the semiconductor, leading to an optimal device performance.^42^ The large VBO between the GaN and n-GaInP is beneficial as it leads to a selective transport of electrons and prevents interfacial nonradiative recombination, leading to a higher photovoltage and performance.^65,79−81^ By facilitating electron transport while hindering hole transport, GaN may be a promising candidate for a charge-selective contact and passivation layer for the PEC device application.^71,82,83^

## Conclusions

The investigation of the GaN/oxide/n-GaInP heterostructure using photoelectron spectroscopy has provided valuable insights into the alignment of its electronic band structure across the heterostructure and shows potential benefits of GaN as a passivation layer for PEC devices. Through a comprehensive analysis of the MOVPE-prepared P-rich n-GaInP(100) surface, the oxidized n-GaInP surface, and ALD-deposited GaN layers, we successfully derived the band alignment of this complex heterostructure.

Our study revealed a type-II heterostructure with a VBO of about 2.01 eV between the oxide layer and the n-GaInP epilayer and a VBO of 1.90 eV (CBO of 0.44 eV) between the oxidized n-GaInP epilayer and the GaN passivation layer. Additionally, we observed evidence of changes in charged states at the surface and, accordingly, changes in surface BB at the oxide/n-GaInP and GaN/oxide/n-GaInP heterointerfaces. Such a band alignment with low CBO, and high VBO, forms a nearly barrier-free pathway across the interface for the electrons, which is ideal for the selective transport of electrons on the cathode side in PEC devices. Despite the presence of a barrier attributed to the oxide layer, its thinness permits electron tunneling, facilitating electron transport across the interface. It is worth considering that exploring the effects of thinning the oxide layer could be a valuable next step. This could help improve the way electrons move across the interface and improve the overall performance of the devices.

## Supporting Information Available

Experimental results of doping concentration measured by ECVP; XRD measurements of the GaInP(100) lattice matched to GaAs; XRD measurements of the GaN layer to study the crystallinity; schematic of the sequence of each ALD cycle; XP spectra of C 1s and O 1s before and after etching; calculations of thickness determination of the oxide layer; atomic percentages of carbon and oxygen content in all samples; and XPS CLs with fitted data of 1, 5, and 10 nm GaN grown on n-GaInP(100) samples (PDF)
