# Supplementary material for: Photoemission Study of GaN Passivation Layers and Band Alignment at GaInP(100) Heterointerfaces
Source: ACS Appl Mater Interfaces. 2025 Jan 14;17(4):7087–97. doi: 10.1021/acsami.4c17453 (PMC11788992; doi:10.1021/acsami.4c17453)
Supplement: Supplementary file 1 — am4c17453_si_001.pdf [file am4c17453_si_001.pdf]

Supplementary Material

Photoemission study of GaN passivation layers and band alignment at GaInP(100) heterointerfaces

Sahar Shekarabi,[1] Mohammad Amin Zare Pour,[1] Haoqing Su,[2] Wentao Zhang,[2] Chengxing He,[2] Kai Daniel Hanke,[1] Oleksandr Romanyuk,[3] Agnieszka Paszuk,[1] Wolfram Jaegermann,[4] Shu Hu,[*,2] and Thomas Hannappel[*,1]

[1] Fundamentals of Energy Materials, Institute of Physics, Technische Universität Ilmenau, 98693 Ilmenau, Germany

[2] Department of Chemical and Environmental Engineering, Yale University, New Haven, CT 06520, USA

[3] FZU – Institute of Physics of the Czech Academy of Sciences, Prague 16200, Czech Republic

[4] Surface Science Laboratory, Department of Materials and Earth Sciences, Technische Universität Darmstadt, 64287 Darmstadt, Germany

E-mail: shu.hu@yaho.edu, Thomas.Hannappel@tu-ilmenau.de

Doping level measured by ECVP:

Electrochemical capacitance voltage profiling (ECVP, WEP-CVP 21) was employed to conduct a depth profile analysis of carrier concentration. The etching solution used was 1 M HCl. The surface of the sample yielded a carrier concentration of 2.0 E16 cm^{-3}. Notably, the interface between the GaAs buffer layer and GaInP exhibited a carrier concentration of approximately 2.0 E17, indicating the occurrence of silicon diffusion from the GaAs layer into the GaInP layer.

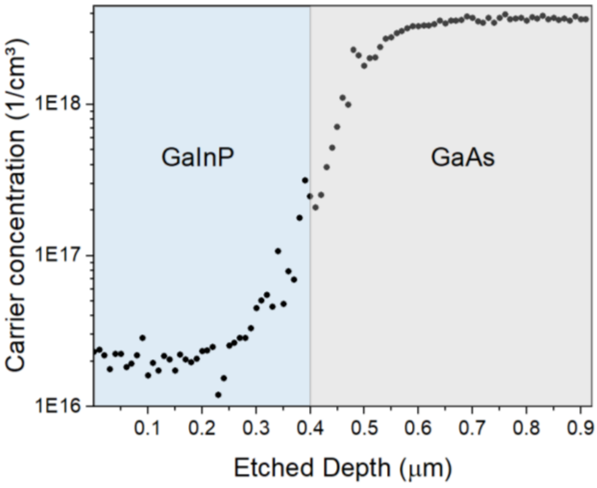

Figure S1: Carrier concentration profiles obtained on 400 nm GaInP sample by CVP.

Study lattice matching by XRD measurement:

High-resolution X-ray diffraction (XRD) ω/2θ scans of GaAs(004) reflection measured by Bruker AXS D8 Discover diffractometer. Fig. S1 displays diffraction intensity of 400 nm GaInP on GaAs(100). Diffraction peak maxima lies at 33.04°. Which confirms the identical lattice constant across the overlayer and the GaAs(100) substrate. Moreover, the derived In atomic stoichiometry is x=0.51 in $Ga_xIn_{1-x}P$ [1].

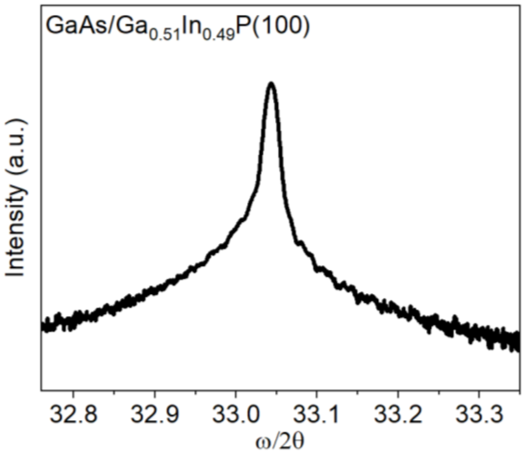

Figure S2: XRD scan of GaAs(004) reflection from 400 nm GaInP on GaAs(100) heterostructure.

Study the crystallinity of the GaN layer:

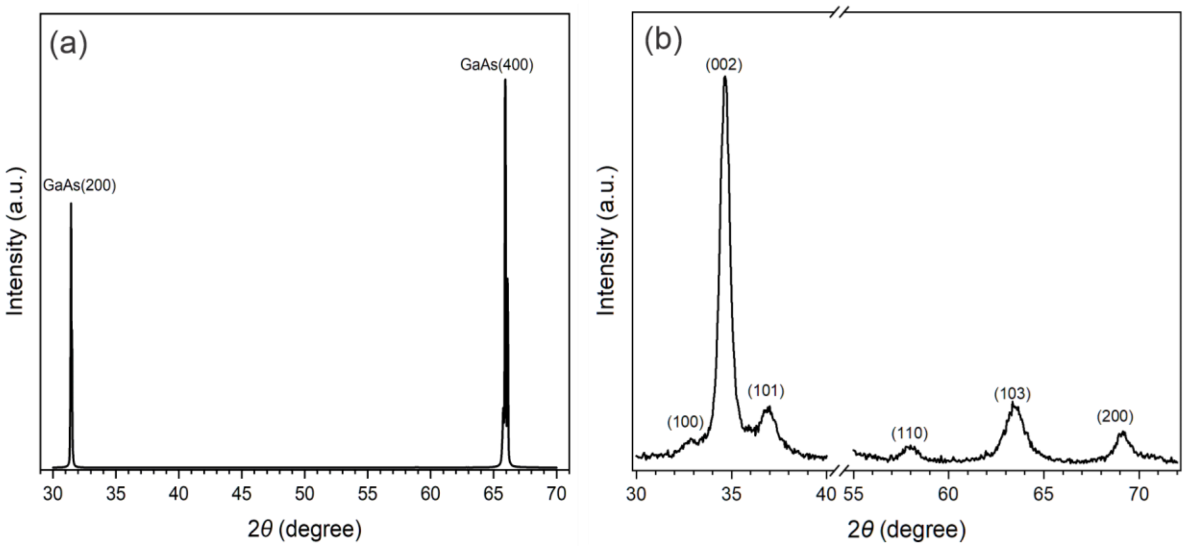

Figure S3: (a) θ-2θ scan results for the 30 nm ALD-grown GaN on a GaInP/GaAs substrate, with the (100) edge parallel to the X-ray beam plane, showing diffraction peaks from the GaAs and GaInP layers. (b) Grazing incidence X-ray diffraction (GIXRD) spectra at an incident angle of ω=0.313°, highlighting the diffraction pattern from various planes of the GaN coating, indicating a well-defined polycrystalline structure.

ALD growth:

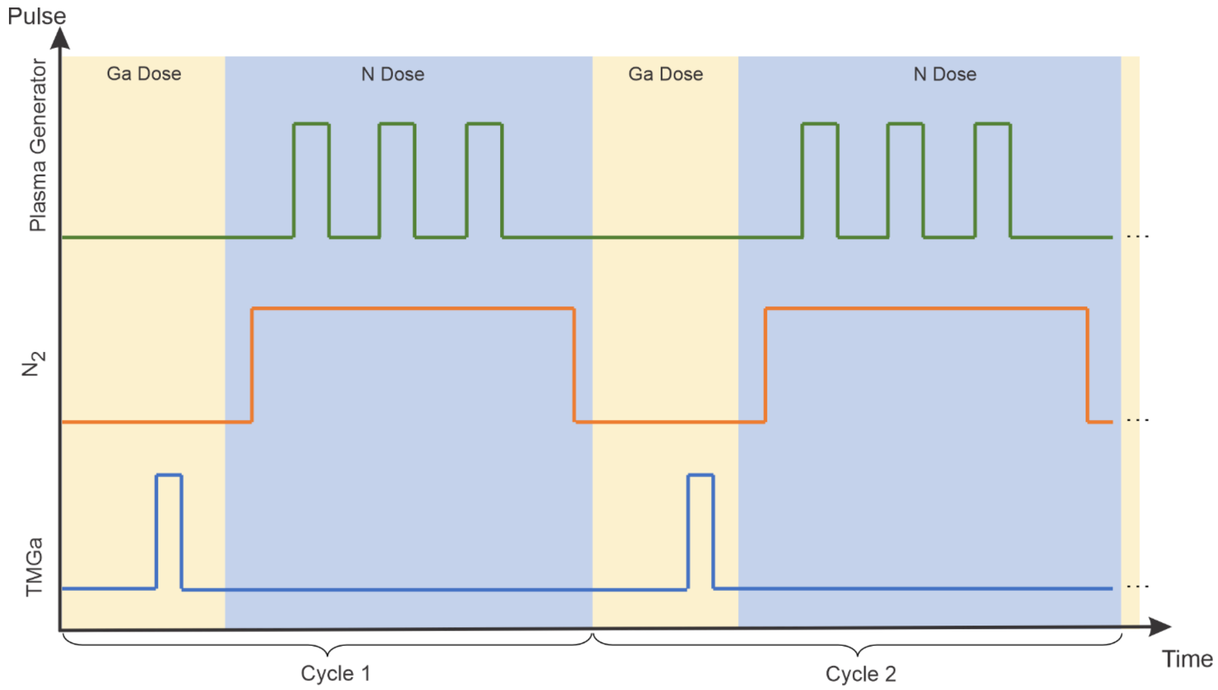

Figure S4: Sequence of each ALD cycle, which comprises a Ga dose followed by an N dose, with 30 sccm of Ar flow serving as the carrier gas throughout the process. During the Ga dose, a 0.015 second pulse of TMGa is introduced with 80 sccm of Ar and 5 sccm of N_2 directed through the plasma generator, with both the generator and the turbo pump deactivated. In the subsequent 60-second N_2 dose, the turbo pump is activated to connect to the reaction chamber, with 200 sccm of Ar and 40 sccm of N_2 flowing through the plasma generator. The plasma generator is then activated three times, each time at 300 W for 20 seconds. The process pressures are maintained at 0.16 mbar during the Ga dose and 0.04 mbar during the N dose, reflecting the changes in plasma flow rate and turbo pump status.

XP-spectra after etching:

For a duration of one minute, the surface of the n-GaInP exposed to air was treated with a commercial solution containing 1.25 M of HCl dissolved in 2-propanol (Aldrich) to reduce surface contaminants.

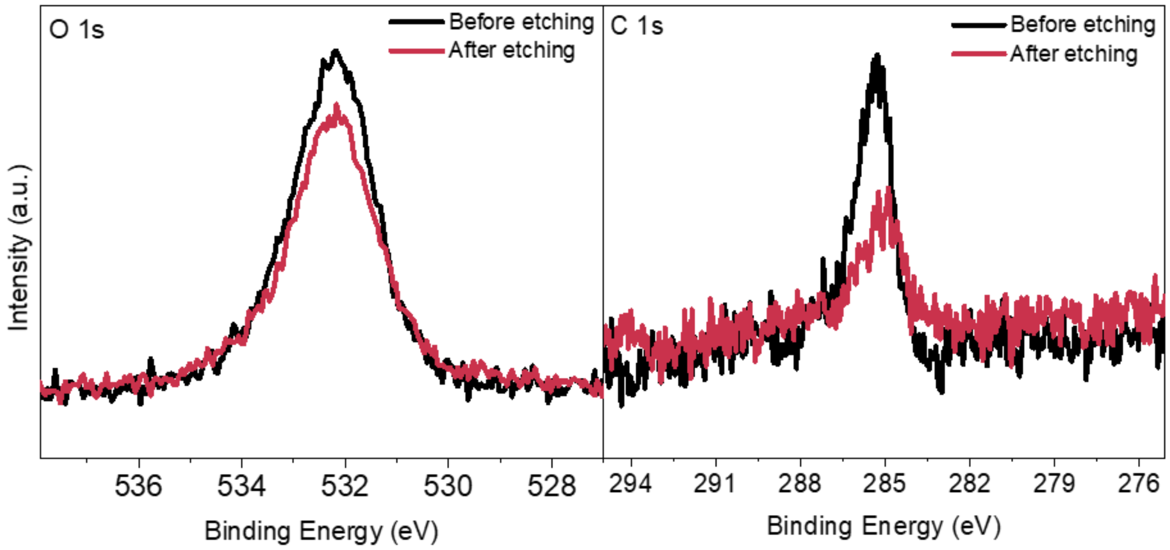

Figure S5: Measured core level spectra of O 1s (left) and C 1s (right) of n-GaInP before and after etching.

Thickness of the oxide layer:

To evaluate the oxide thickness, following equation:

$$d = \lambda \ln (Io/Ib + 1) \qquad \text{Eq. S1}$$

was employed. By considering the electron attenuation length, written as λ, to be 2.3 nm [2], and the intensities of oxide and bulk are denoted I_o and I_b, respectively. The resulting oxide layer thickness is around 1-5 Å [3].

Table. S1: Calculated oxide layer thickness

Thickness	Core levels	Intensity of oxide (I_O)	Intensity of bulk (I_b)	Thickness of oxide (Å)
0 nm	P 2p	1518.61	6285.28	4.90
	In $3d_{5/2}$	1924.58	31834.93	1.30
	Ga $2p_{3/2}$	5765.70	31886.91	3.80

Atomic percentage of carbon and oxygen:

The atomic percentage of carbon and oxygen with respect to GaN overlayer thickness is shown in Fig. S6 which were derived from the integrated core level peak intensities with the respective sensitivity factors.

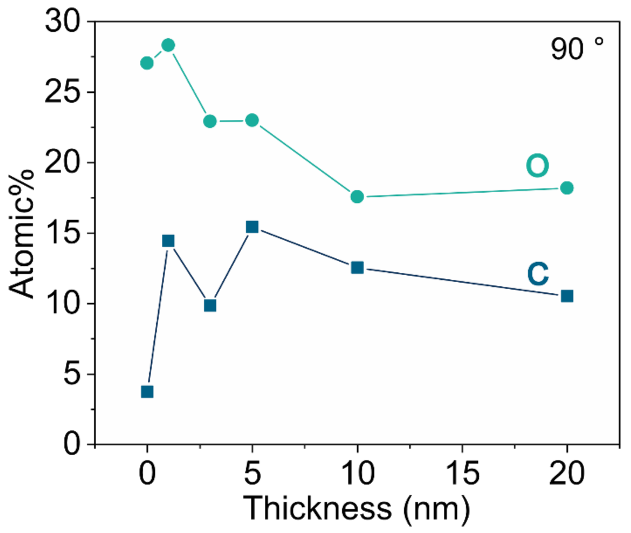

Figure S6: The atomic concentration of Oxygen and Carbon of n-GaInP/GaN samples for 0-20 nm overlayer thickness.

Fittings of core levels of 1, 5 and 10 nm GaN/n-GaInP samples:

The Ga $2p_{3/2}$ peak contains intensity contributions originating from various sources, including the GaN overlayer (Ga-N component), the underlying n-GaInP substrate (Ga-P component), as well as contributions stemming from oxide and Ga-Ga bonds. Within the In $3d_{5/2}$ peak, distinct components are discerned, notably a component associated with In-P bonds and additional features attributable to In-O_x interactions. In the N 1s spectra, a range of contributions arise from N-Ga bonds, Ga LMM Auger peaks, and N-O_x bonds. Notably, a consistent binding energy difference between the positions of the Ga-N related component in the Ga $2p_{3/2}$ and N 1s peaks is observed across all samples, averaging 720.38 ± 0.19 eV. For the P 2p core level, the data is fitted with spin-orbit pairs with the same FWHM and a peak ratio of 2:1, along with a splitting of 0.84 eV. The main component at around 129 eV corresponded to the dominant bulk P-III interaction, the small feature around 130 eV (orange peak), correlated to the surface InP_x/P-P or P-Cl components. Additionally, the emergence of a third component around 134 eV

indicated the presence of PO_x species. There were no In or P atom contributions observed for 10 nm GaN sample.

One of the crucial oxide species in PEC application is In_2O_3 with a band gap of 2.90 eV. This oxide species serves as a trap for charge carriers. The presence of this species is typically observed in the O 1s core level at an energy of 530 eV. According to the O 1s core level (k), in oxidized GaInP sample (0 nm), In_2O_3 is resolved, but we can see by deposition of GaN this type of oxide is reducing. And because of that we did not consider any conduction band state for that in the band diagram.

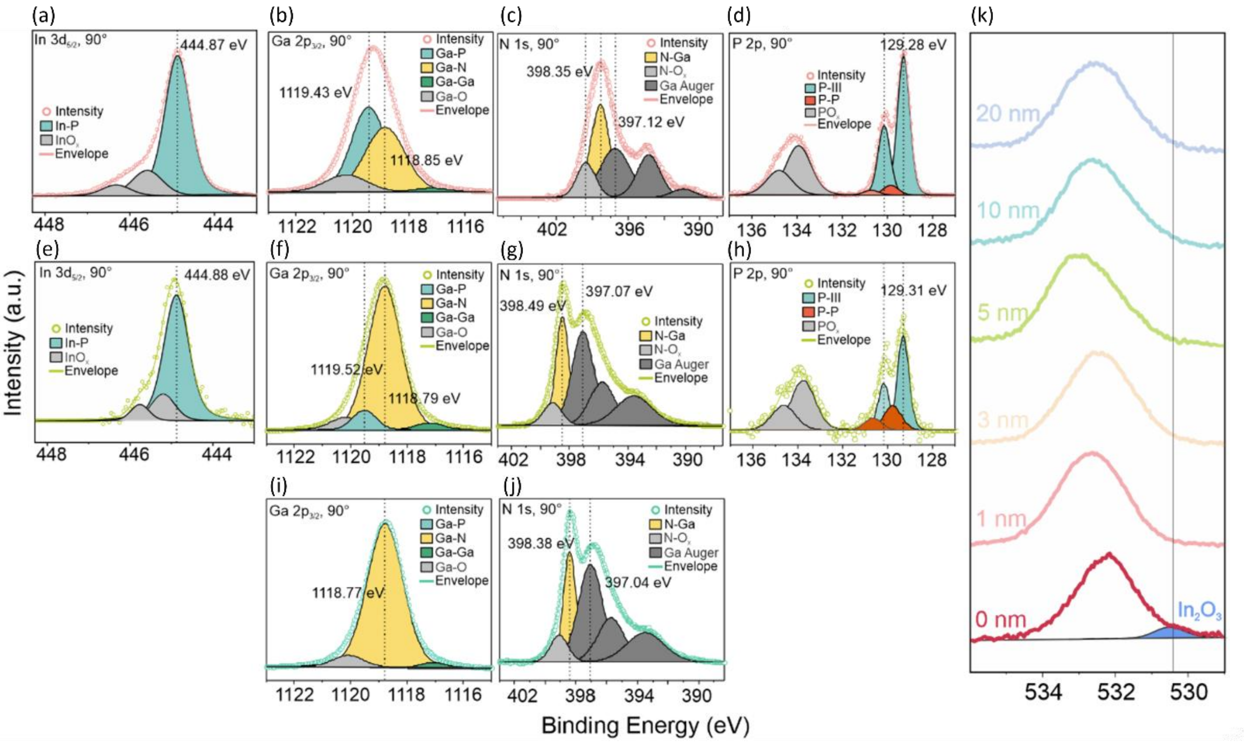

Figure S7: XP spectra of a-d) 1nm GaN/n-GaInP, e-h) 5 nm GaN/n-GaInP and i-j) 10 nm GaN/n-GaInP, and k) O 1s spectra of 0 to 20 nm GaN/n-GaInP. All spectra are shown after background subtraction.

Supplementary References

[1] Miller, A. M., Lemon, M., Choffel, M. A., Rich, S. R., Harvel, F., & Johnson, D. C. (2022). Zeitschrift für Naturforschung B, 77(4-5), 313-322.

[2] Powell, C., Jablonski, A. (2011). NIST Electron Effective-Attenuation-Length Database, Version 1.3, Standard Reference Data Program Database 82, National Institute of Standards and Technology, Gaithersburg, MD. URL: http://www.nist.gov/srd/nist82.cfm.

[3] Briggs, D., Seah, M. P. (1990). Practical Surface Analysis, Auger and X-Ray Photoelectron Spectroscopy, 1, 151-152.
